# Combination treatment with FAAH inhibitors/URB597 and ferroptosis inducers significantly decreases the growth and metastasis of renal cell carcinoma cells via the PI3K-AKT signaling pathway

**DOI:** 10.1038/s41419-023-05779-z

**Published:** 2023-04-06

**Authors:** Junfeng Hao, Qiguang Chen, Yongmin Feng, Qiyu Jiang, Huiwei Sun, Botian Deng, Xin Huang, Jibin Guan, Qiuping Chen, Xincheng Liu, Yanjin Wang, Peng Cao, Fan Feng, Xiaoyu Li

**Affiliations:** 1 Department of Nephrology, and Guangdong Provincial Key Laboratory of Autophagy and Major Chronic Non-communicable Diseases, Affiliated Hospital of Guangdong Medical University, Zhanjiang, 524001 China; 2 Department of General practice medicine, Shengjing Hospital of China Medical University, Shenyang, 110022 China; 3 Department of Urology, The First Affiliated Hospital of China Medical University, Shenyang, 110001 China; 4 Department of Infectious Diseases, Fifth Medical Center of Chinese PLA General Hospital, Institute of Infectious Diseases, Beijing, China; 5 Masonic Cancer Center, University of Minnesota, Minneapolis, MN 55455 USA; 6 Department of Geriatrics, Affiliated Hospital of Guangdong Medical University, Zhanjiang, 524001 China; 7 Department of Neurosurgery, General Hospital of Northern Theater Command, 83 Wenhua Road, Shenyang, 110016 China; 8 Clinical Laboratory, The Fifth Medical Center of Chinese People’s Liberation Army General Hospital, Beijing, China

**Keywords:** Oncogenes, Renal cell carcinoma

## Abstract

Ferroptosis, a nonapoptotic form of programmed cell death characterized by significant iron-dependent peroxidation of phospholipids, is regulated by cellular metabolism, redox homeostasis, and various cancer-related signaling pathways. Recently, considerable progress has been made in demonstrating the critical role of lipid metabolism in regulating ferroptosis, indicating the potential of combinational strategies for treating cancer in the future. In this study, we explored the combinational effects of lipid metabolism compounds and ferroptosis inducers on renal cell carcinoma (RCC) cells. We found potent synergy of the fatty acid amide hydrolase (FAAH) inhibitor URB597 with ferroptosis inducer (1S, 3R)-RSL3 (RSL3) in inhibiting the growth and metastasis of RCC cells both in vitro and in vivo via induction of G1 cell cycle arrest and promotion of the production of lipid peroxides, malondialdehyde (MDA), 4-hydroxynonenal (4-HNE), and cytosolic reactive oxygen species (ROS). In addition, inhibition of FAAH increased the sensitivity of RCC cells to ferroptosis. Genome-wide RNA sequencing indicated that the combination of URB597 and RSL3 has more significant effects on regulation of the expression of genes related to cell proliferation, the cell cycle, cell migration and invasion, and ferroptosis than either single agent alone. Moreover, we found that combinational treatment modulated the sensitivity of RCC cells to ferroptosis via the phosphatidylinositol 3 kinase (PI3K)-AKT signaling pathway. These data demonstrate that dual targeting of FAAH and ferroptosis could be a promising strategy for treating RCC.

## Introduction

Renal cell carcinoma (RCC) is the most common type of kidney cancer in adults, responsible for ~90–95% of kidney malignancies [1–3]. Surgery is the most effective treatment for RCC, but up to 30% of newly diagnosed patients develop metastasis (with a 5-year survival rate of 10%), and 20–30% post-surgery treatment cases eventually experience a recurrence [4–6]. Because RCC is resistant to traditional chemotherapy, hormonal therapy, or radiation therapy, investigation of the molecular mechanisms underlying RCC tumorigenesis and potential therapies is crucial for individual treatment of RCC [6–8]. In particular, RCC is generally accompanied by a reprogramming of glucose, fatty acid, amino acid, and glutathione metabolism [9, 10], and these changes provide opportunities for new biomarkers and potential strategies to be used to treat RCC, especially when metastasis has limited long‑term treatment options.

Ferroptosis is a type of programmed cell death different from apoptosis, necrosis, or pyroptosis [11, 12]. Its biochemical characteristics include the accumulation of intracellular iron ions and lipid peroxides [13]. The hallmark of ferroptosis is a lethal accumulation of lipid peroxides caused by the oxidation of phospholipids containing polyunsaturated fatty acids, which is a form of disordered metabolism of intracellular lipid oxides, and ferroptosis is associated with a variety of biological processes and is regulated by signaling pathways, including cellular metabolism and redox homeostasis [14–16]. Increasing studies have classified that pivotal role of ferroptosis in the occurrence and development of neurodegenerative diseases, infections, and inflammatory diseases and carcinogenesis [17–19]. In recent years, the inducing of ferroptosis in tumor cells has become a potential strategy for tumor therapy by modulating various tumor properties, and various ferroptosis inducing agents or drugs such as sulfasalazine, erastin, RSL3, sorafeinib have been developed in reducing cancer growth and resistance [20–22]. Nevertheless, the unsatisfactory antitumor pharmacological effect in vivo of ferroptosis inducers single agent and low selectivity for normal cell types limit the application of ferroptosis in clinical cancer therapy, which motivating novel strategies [11].

Abnormal lipid metabolism is closely related to sensitivity to ferroptosis, and lipid metabolism plays an important role in the regulation of cancerous cells by inducing or inhibiting ferroptosis in tumor cells. This indicates that combination therapy targeting lipid metabolism and ferroptosis is a promising cancer therapy [23–25]. In this process, Glutathione Peroxidase 4 (GPX4) is the most important regulator of iron death-related mechanisms, and RSL3, as a small-molecule inhibitor of GPX4, is currently the most commonly used Ferroptosis inducer [20–25]. Moreover, the endocannabinoid system exerts anticarcinogenic effects via multiple mechanisms, including proapoptotic and antiproliferative properties [26–28]. Fatty acid amide hydrolase (FAAH), a member of the serine hydrolase family of enzymes, was first identified as the principal catabolic enzyme that catalyzes the hydrolysis of endogenous amidated lipids such as N arachidonoylethanolamine (AEA, anandamide), palmitoylethanolamide (PEA), and oleoyl-ethanolamide (OEA), which regulating various metabolic pathways and pathophysiological conditions in the body, such as emotion, cognition, energy balance, pain sensation, neuroinflammation, and cancer cell proliferation [29–31]. FAAH is upregulated in a variety of tumors, such as prostate cancer, and its inhibition results in similar antiproliferative and antimetastatic effects [32–34]. FAAH inhibitors confer anti-invasive and antimetastatic effects on various cancer cells and also have synergistic effects with chemotherapeutic drugs [35–37]. Several nonselective and selective inhibitors of FAAH, including URB597, a relatively selective, irreversible, carbamate-based inhibitor, exert potential anticancer effects in ovarian, breast, prostate, and colorectal cancers [38–40], suggesting that targeting FAAH might be a promising anticancer strategy. Although it is widely established that FAAH has diverse effects contributing to tumor progression, its effect on ferroptosis has not ever been explored.

Here we show that by working in combination URB597 (a typical inhibitor of FAAH) and RSL3 significantly decrease the growth, migration, and invasion of RCC cells in vitro and in vivo via increasing lipid peroxidation, increasing the production of cellular reactive oxygen species (ROS), as well as inducing cell cycle arrest. Results through analysis, the interaction between the two is synergistic. Consistent with these synergic anticancer effects, genome-wide RNA sequencing (RNA-Seq) indicates that the combination of URB597 and RSL3 has more significant effects on regulation of the expression of genes related to cell proliferation, cell cycle, cell migration, and invasion as well as ferroptosis than monotherapy. These findings suggest that dual targeting of FAAH and ferroptosis could be a promising therapeutic strategy for inhibiting RCC growth and metastasis.

## Materials and methods

### Patients

Patients (*N* = 80) with advanced RCC were admitted to the Department of Urology of The First Affiliated Hospital of China Medical University from April 2015 to December 2019. The collection and use of patient tumor tissue and other clinical specimens were reviewed and approved by the Medical Ethics Committee of The First Affiliated Hospital of China Medical University. Patient tissue samples were derived from tumor tissue samples obtained during the course of normal treatment (including surgical resection or minimally invasive treatment) with histologically or cytologically confirmed RCC with a clear cell component. Patients had an Eastern Cooperative Oncology Group performance status of 0 or 1 and a life expectancy of 12 weeks or more. Patients were classified by MSKCC (Memorial Sloan-Kettering Cancer Center) risk group: favorable (no factors), intermediate (one factor), or poor (two or three factors). Quantitative polymerase chain reaction was used to detect the expression of GPX4 (the target of RSL3) and FAAH in tumor tissue; patients were divided into groups according to the median expression of GPX4 (GPX4-high group/GPX4-low group) or FAAH (FAAH-high group/FAAH-low group). Patients were then followed to determine differences in overall survival between the groups. Results are shown as survival curves, median survival, and 95% confidence intervals and P values. For the sample size estimate, the sample size (80 RCC specimens) used in the presence work has an adequate power to detect a pre-specified effect size (the 1 − *β*: 0.8; *α*/2: 0.025; *P* < 0.05) [41].

### Correlation analysis

In 80 tumor tissues, mRNA expression of GPX4 and FAAH, respectively, was detected, and a scatter plots were drawn with GPX4 expression as the abscissa and FAAH expression as the ordinate. Each tumor tissue corresponded to a data point. Linear regression was performed on the data point group, and the regression equation of the correlation between GPX4 and FAAH was obtained. The *p* value of an equation whose slope is not 0 indicates whether there is a correlation between the expression of GPX4 and FAAH: A positive slope indicates a positive correlation, and a negative slope indicates a negative correlation.

### Chemicals and drugs

All small-molecule inhibitors were purchased from Targetmol (Wellesley Hills, MA, USA), including FAAH inhibitors (FAAH-IN-2, URB597, PF-3845, JNJ-42165279, BIA 10-2474, FAAH-IN-1, SA47, LY-2183240, FAAH inhibitor 1, JNJ-1661010, PF-04457845, 4-nonylphenylboronic acid, N-benzylpalmitamide, SA72, JZL184, JZL195), ferroptosis inducers (erastin, T1765; RSL3, T3646; ML-210, T8375; FIN56, T4066; CIL56, T4309; iFSP1; sorafenib; BAY 87-2243; sulfasalazine; ML162), PI3K inhibitor (LY294002, T2008), and MEK inhibitor (PD98059, T2623). These drugs were prepared as formulations for cell-based assays or the animal according the methods descripted in the previous publications [42–45].

### Cell lines

All cell lines used in this study were purchased from the American Type Culture Collection (Manassas, VA, USA) and were authenticated according to the short tandem repeat profile. Human embryonic kidney 293T cells were grown in Dulbecco’s modified Eagle medium (Hyclone, Logan, UT, USA) containing 25 mmol/L glucose (Invitrogen, Carlsbad, CA, USA), whereas RCC cell line (Caki-1, 786-O, OS-RC-2, SW839, GRC-1) cells were grown in Roswell Park Memorial Institute medium (Hyclone). RCC cell line G-401 cells were grown in McCoy’s 5 A medium (Hyclone). RCC cell line A498 was grown in Eagle’s minimum essential medium (Gibco, Grand Island, NY, USA). All cell lines were cultured in medium containing 10% fetal bovine serum (Hyclone), 1% penicillin (Invitrogen), and 1% streptomycin (Invitrogen). All cells were cultured at 37 °C and 5% CO_2_.

### Antibodies

Rabbit anti-β-actin (20536-1-AP), rabbit FAAH (17909-1-AP), rabbit anti-Ki67 (27309-1-AP), and mouse GPX4 (67763-1-Ig) were purchased from Proteintech (Rosemont, IL, USA). Anti-4-HNE (ab46545) was purchased from Abcam (Boston, MA, USA).

### Plasmids and shRNA lentivirus infection

Lentiviral constructs of human FAAH shRNAs were purchased from GeneChem (Shanghai, China). Lipofectamine 3000 reagent was used for the transfection of plasmids according to the manufacturer’s instructions (Invitrogen). For shRNA lentivirus infection, lentivirus was generated by transfection of the 293T producer cell line with the lentiviral vector and packing vector mix (System Biosciences, USA). Lentivirus was collected 48 h later and was used to infect 786-O and Caki-1 cells. Stable cell lines were selected with puromycin (3 µg/mL) 48 h after infection. Pooled clones were screened by western blotting with anti-FAAH. For plasmid transfection, cells were seeded to 70–90% confluent at the time of transfection. Plasmids and P3000 reagent were diluted in Opti-MEM (Invitrogen). The diluted plasmids were mixed with the diluted Lipofectamine 3000. The mixtures were incubated for 15 min at room temperature (25–30 °C), and the mixture was added to cells in each dish. The transfected cells were collected after 24–48 h.

### Drug combinational screening and drug synergy assay

786-O cells were seeded in 96-well plates for each cell line at 4000 cells per well, and a cell viability assay was conducted with a CCK-8 kit (Dojindo Laboratories, Japan) after combinational treatment with FAAH inhibitors and ferroptosis inducers for 72 h according to the manufacturer’s instructions. For the drug synergy assay, the synergic effect of drug pairs was defined quantitatively with the Chou–Talalay equation [46], where a combination index < 1 indicates synergism and a combination index > 1 indicates antagonism. 786-O and Caki-1 cells were treated with different concentrations of two single drugs and combinational treatment, respectively, for 72 h, and cell viability was validated with a CCK-8 kit (Dojindo Laboratories) according to the manufacturer’s instructions.

### Cell viability and colony formation assays

For the cell viability assay, RCC cells were seeded in 96-well plates (100 µL per well) at 5000 cells/well to adhere overnight and then treated with the described drug doses for 72 h. Dimethyl sulfoxide was used as a control. Anchorage-dependent cell viability was evaluated with a CCK-8 kit (Dojindo Laboratories) according to the manufacturer’s instructions. For the colony formation assay, stably transfected 786-O and Caki-1 cells or parental 786-O and Caki-1 cancer cells were plated in six dishes in triplicate at 2000 cells per well to adhere overnight and then treated with the described drug doses for 10–14 days. During the drug treatment, the cell medium containing the drug was replaced every 3 days. Subsequently, colonies were fixed with 4% paraformaldehyde for 25 min and stained with 0.2% crystal violet solution for 30 min at room temperature (25–30 °C). The cells were then washed, dried, and scanned with an HP Scanjet.

### Cell migration and invasion assays

For the cell migration assay, confluent monolayers of cells were scratched mechanically with a 200-µL pipette tip. The debris was washed three times with phosphate-buffered saline (PBS) and treated with dimethyl sulfoxide or compounds in Dulbecco’s modified Eagle medium (high glucose) without fetal bovine serum accompanied by mitomycin C (1 µM) treatment. Cells were photographed at 0 h and 12 h from the same position, and the number of cells that had migrated was counted. For the cell invasion assay, 10 mL liquid Matrigel (BD Biosciences) melted on ice was added dropwise to the upper surface of a transwell chamber (Corning). Cancer cells were washed three times with PBS and added to each well with dimethyl sulfoxide or compounds treatment-containing medium (the drug-treamnet group) at 10,000 cells per well accompanied by mitomycin C (1 µM) treatment. After 24 h, 4% paraformaldehyde was used to fix the cells that had invaded the matrix gel membrane, and then the cells were stained with crystal violet. Photographs were taken, and the number of invading cells was counted.

### Measurement of lipid peroxidation

C11-BODIPY dye (10 µM; D3861; Thermo Fisher Scientific, Waltham, MA, USA) was used for lipid ROS staining according to the manufacturer’s instructions, with cumene hydroperoxide as a positive control. Briefly, stably transfected or parental 786-O and Caki-1 cancer cells were collected after treatment with URB597 and RSL3 singly or in combination (URB597 + RSL3) for 2 days and 10 µM C11-BODIPY-containing medium for 1 h. The lipid ROS level was determined by flow cytometry analysis (FACS Canto II; BD Biosciences).

### Measurement of ROS

786-O and Caki-1 cells (1 × 10^6^ cells) were cultured in six-well dishes to adhere overnight and then treated with compound for 48 h. Subsequently, the cell culture medium was removed and 2 mL diluted 25 µM carboxy-H_2_DCFDA (88-5930-74; Invitrogen) was added to the six-well plate, followed by incubation at 37 °C for 30 min. Cells were washed gently three times with warm PBS, and tert-butyl hydroperoxide was added to the positive control well. ROS levels were determined by flow cytometry analysis with a 488 nm excitation wavelength and a 525-nm emission wavelength (FACS Canto II; BD Biosciences).

### Evaluation of malondialdehyde (MDA) and 4-hydroxynonenal (4-HNE) levels

The relative concentration of MDA in cell or tumor lysates was assessed with a Lipid Peroxidation (MDA) Assay Kit (ab118970; Abcam) according to the manufacturer’s instructions. This assay measures the reaction of MDA with thiobarbituric acid (TBA), which generates an MDA-TBA adduct. The MDA-TBA adduct can be quantified colorimetrically (OD = 532 nm). A Lipid Peroxidation (4-HNE) Assay Kit (ab238538; Abcam) was used to evaluate the concentration of 4-HNE according to the manufacturer’s protocol.

### Cell cycle analysis

786-O and Caki-1 cancer cells (1 × 10^6^ cells) were cultured in six-well dishes to adhere overnight and then treated with URB597 and RSL3 singly or in combination (URB597 + RSL3) for 48 h. For the cell cycle analysis, cells were fixed in 75% ethanol for more than 18 h at 4 °C, washed with PBS, and incubated with RNase A (0.2 mg/mL) in PBS. Propidium iodide was then added to the cell suspension. Samples were analyzed with a FACS Calibur Flow Cytometer (Becton Dickinson, Franklin Lakes, NJ, USA) [47–49].

### Quantitative reverse-transcription PCR (RT-qPCR)

Samples were homogenized with TRIzol reagent (Invitrogen), vortexed for 1 min with 200 mL chloroform, and centrifuged at 1.2 × 10^4^ rpm for 15 min at 4 °C. The upper aqueous phase (containing RNA) was precipitated with 400 µL isopropanol at room temperature for 10 min and centrifuged at 1.2 × 10^4^ rpm for 10 min at 4 °C. The RNA pellets were washed with 70% (v/v) ethanol and then washed with 100% (v/v) ethanol. Then the RNA pellets were air-dried and dissolved in 50 µL nuclease-free water. Finally, 2 mg total RNA reverse-transcribed to cDNA according to the manufacturer’s recommendation (Takara, Japan).

### Western blot

786-O and Caki-1 cells were collected and washed with PBS and lysed in RIPA buffer. The protein concentration was quantified and total protein lysates were separated by 10% SDS-PAGE and transferred to nitrocellulose membrane. Membranes were sequentially probed with indicated primary and secondary antibodies and were imaged by the Imaging system (Bio-rad, Hercules, CA, USA).

### RNA-Seq

A minimum of 3 µg total RNA was oligo (dT) selected using a Dynabeads mRNA purification kit (Invitrogen). A NEBNext® UltraTM RNA Library Prep Kit for Illumina® (NEB, USA) was used to generate a sequencing library. Briefly, mRNA was extracted and then purified from the total RNA. Double-stranded cDNA was synthesized with these short fragments as templates. The cDNA was end-repaired, ligated to Illumina adapters, size-selected on agarose gel (approximately 250 bp), and PCR amplified. The cDNA library was sequenced on an Illumina HiSeq 6000 sequencing platform (BerryGenomics). We estimated gene expression levels for each transcript of exon model as the number of reads per kilobase per million mapped reads using only uniquely mapped reads in exonic regions. A gene was considered significantly differentially expressed if its expression differed between any two samples with a fold change > 2 and *p* < 0.05 as calculated by Cufflinks. The RNA-Seq data are available in the Gene Expression Omnibus database (http://www.ncbi.nlm.nih.gov/geo/) under accession ID.

### Animal models for tumor growth

Nude mice were purchased from Vital River Laboratory Animal Technology (Beijing) and housed in an SPF animal facility. For tumor xenografts, 5 × 10^6^ 786-O-luciferase cells were injected subcutaneously into the axilla of 6-week-old female nude mice. Tumor size was measured at the indicated time using calipers. Tumor volume was estimated according to the following formula/equation: volume = (longest diameter × shortest diameter^2^)/2. When tumors reached a volume of approximately 50 mm^3^, the mice were randomly divided into groups and injected intraperitoneally with URB597 (30 mg/kg) and RSL3 (30 mg/kg); control animals were injected with an equivalent volume of saline. The experiment was terminated when the maximum tumor size reached approximately 1.5 cm in diameter, at which point the tumors were isolated from the animals and weighed. For the metastasis model, 2 × 10^5^ metastasis 786-O-luciferase cells were injected into the tail veins of nude mice. The mice were randomly divided into groups and injected intraperitoneally with URB597 (30 mg/kg) and RSL3 (30 mg/kg); control animals were injected with an equivalent volume of saline. Images of xenograft mice were obtained with a Xenogen IVIS 2000 Luminal Imager once a week. For the animal experiments’ statement about randomization: animals were randomly numbered and randomly assigned to groups based on a random number table method, and there was no subjective preference or subjective influence in the selection of animals for each group.

### Endocannabinoid quantification by HPLC-MS

Endocannabinoid levels in 786-O tumors were quantified by directly homogenizing the tissue in mixture of chloroform-methanol-water 4:2:1 (v/v/v) solution. After mixing and phase separation by centrifugation, the organic layer was recovered and dried under a stream of N2. The resulting lipid extracts were purified by solid-phase extraction using silica and elution with an EtOAc-Acetone (1:1) solution. The resulting lipid fraction was analyzed by HPLC-MS using an LTQ Orbitrap mass spectrometer coupled to an Accela HPLC system (Thermo Fisher Scientific). Analyte separation was achieved using a C-18 Supelguard pre-column and a Supelcosil LC-18 column (3 μM, 4.6 × 150 mm) (SigmaAldrich, Saint Louis, MO, USA). Endocannabinoid levels were quantified by isotope dilution using their respective deuterated standards with identical retention. The data were normalized by tumor sample weight in the in vivo testing.

### Immunohistochemistry

Paraffin sections (3 mm) were mounted on Plus slides and dried in a 60 °C oven. The slides were placed on a Leica BondMax Immunostainer. Antibodies were optimized with a predetermined staining protocol: Ki67, 1:800; 4-HNE, 1:400. Slides were dehydrated and cover-slipped with Cytoseal 60 (Richard-Allan Scientific) mounting medium.

### Ethics statement

The collection and use of clinical specimens was reviewed and approved by the Medical Ethics Committee of The First Affiliated Hospital of China Medical University, Shenyang 110001, China. The use of human-related materials (clinical specimens and cell lines), the experimental design, and the protocols and methods in the present work were reviewed and approved by the ethics review organization (i.e., the Medical Ethics Committee of Affiliated Hospital of Guangdong Medical University). All experiments were performed according to Helsinki Declaration (World Health Organization) guidelines with the written consent of patients.

The animal experiments (including animal breeding, animal welfare, experimental designs, and protocols/methods) were reviewed and approved by the Animal Ethics Committee of China Medical University, Shenyang 110001, China. All animal experiments were performed in accordance with the UK Animals (Scientific Procedures) Act of 1986 and the associated guidelines.

### Statistical analysis

All in vitro experiments were performed in triplicate. Differences between variables were assessed with chi-square analysis, two-tailed Student’s *t* test, or one-way analysis of variance. SPSS 13.0 or Prism 6 (GraphPad) was used to perform all statistical analyses. Data are expressed as means ± standard deviations. In all assays, *p* < 0.05 was considered statistically significant.

## Results

### Correlations between FAAH and ferroptosis genes in cancer samples

**Fig. 1:**
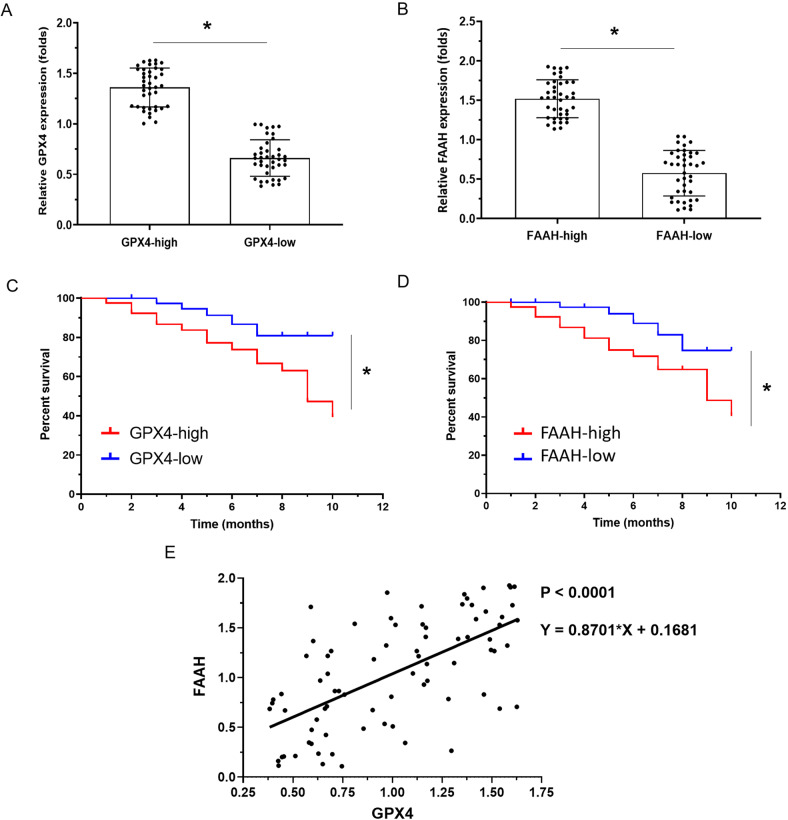
The clinical significance of FAAH and GPX4 in advanced ccRCC. Quantitative polymerase chain reaction was used to detect the expression of FAAH and GPX4 in clinical specimens from ccRCC patients. (**A**, **B**) Patients were divided into two groups (high-expression group or low-expression group) according to their median expression of GPX4 (**A**) and FAAH (**B**). The results are displayed as scatter plots and histograms. Clinical analysis was performed based on follow-up data from patients (**C**, **D**). The prognosis of patients (overall survival) in the high/low groups of GPX4 (**C**) or high/low groups of FAAH (**D**) is shown as a survival curve. *P < 0.05.

Patients with advanced RCC were divided into two groups (high vs. low expression) according to the median expression of FAAH and GPX4 in tumor tissue (Fig. 1A, B and Table 1) and then followed clinically. The prognosis of patients with high GPX4 expression was worse than that of patients with low GPX4 expression, and OS (overall survival) was significantly shorter for patients with high expression than for patients with low expression (8.0, 6.9–8.8 [M] vs. 9.0, 8.4–9.8 [M], *P* = 0.038; Fig. 1C and Table 2). The prognosis of patients with high FAAH expression was worse than that of patients with low FAAH expression, and OS was significantly shorter for patients with high expression than for patients with low FAAH expression (8.2, 6.4–9.1 [M] vs. 9.5, 8.0–10.8 [M], *P* = 0.044; Fig. 1D and Table 3). The expression of GPX4 in tumor tissues was positively correlated with the expression of FAAH (Y = 0.8701*X + 0.16, *P* < 0.0001; Fig. 1E).

**Table 1:** Baseline information of patients.

Patients (number [*n* = 80])	Characters
Age (median [range])	58 (26–79)
Sex (number [rates])	Male (61 [76.25%])
	Female (19 [23.75%])
ECOG status (number [rates])	0 (43 [53.75%])
	1 (37 [46.25%])
MSKCC risk (number [rates])	Favorable (17 [21.25%])
	Intermediate (35 [43.75%])
	Poor (28 [35.00%])

**Table 2:** GPX4 expression and clinical outcome of advanced RCC patients.

	GPX4 mRNA expression		*P*
	High (*n* = 40)	Low (*n* = 40)	
OS	8.1	9.0	0.038
	6.9–8.8 (M)	8.4–9.8 (M)	

*OS* overall survival.

**Table 3:** FAAH expression and clinical outcome of advanced RCC patients.

	FAAH mRNA expression		*P*
	High (*n* = 40)	Low (*n* = 40)	
OS	8.2	9.5	0.044
	6.4–9.1 (M)	8.0–10.8 (M)	

*OS* overall survival.

### Synergy of FAAH inhibitors with ferroptosis inducers significantly decreases the proliferation and migration of RCC cells

**Fig. 2:**
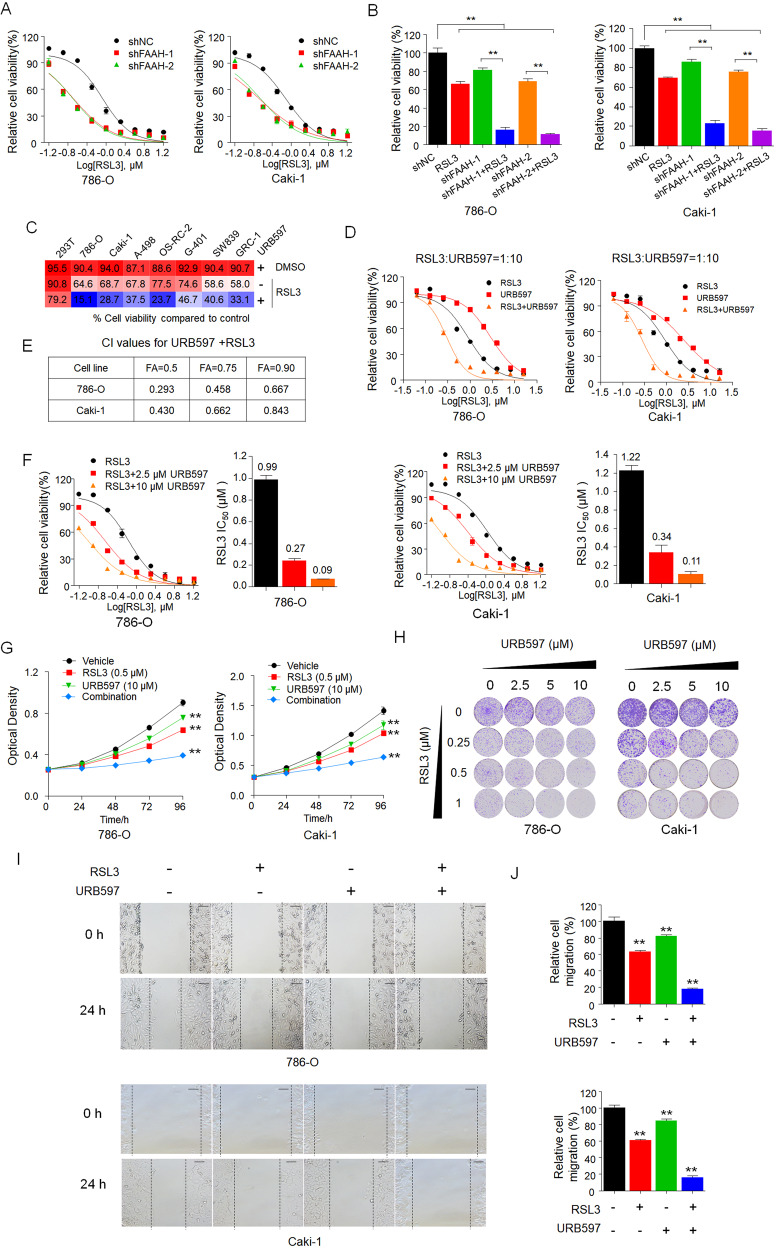
Synergy of URB597 with ferroptosis inducers significantly decreases the proliferation and migration of RCC cells. **A, B** Effect of FAAH knockdown on 786-O and Caki-1 cells in response to RSL3 treatment with two distinct FAAH shRNA expression vectors. Dose–response curves for 786-O and Caki-1 cells stably transfected with negative control vector or shFAAH treated with RSL3 at the indicated doses (**A**). Cell viability was assessed by CCK-8 assay (**B**). **p < 0.01 (one-way ANOVA). **C** Combinational effects of URB597 (10 µM) and RSL3 at the indicated concentrations in various renal cell lines. Cells were treated with the inhibitors singly or the indicated target pairs. Viability was measured 72 h after treatment with the indicated concentrations of drugs. Effects on cell viability were calculated as the percentage of vehicle-treated cells. **D, E** Effects of RSL3 with URB597 singly or in combination in the indicated cell lines. The viability of 786-O and Caki-1 cells was measured 72 h after treatment with the indicated doses of drugs (**D**). The combination index (CI) was calculated with the Chou–Talalay equation using multiple doses and response points. CI values for three different indicated Fa are shown (**E**). **F** Dose–response curves for RSL3 as a single agent or in combination with URB597 at the indicated doses in 786-O and Caki-1 cells for 72 h. The effects of URB597 on the IC_50_ of RSL3 are shown in the bar graphs (right). **G** Proliferation curves of single agents and combinations of drugs in 786-O and Caki-1 cells treated with the indicated concentrations of RSL3 and URB597 for 96 h. Cell viability was assessed by CCK-8 assay after treatment with the indicated doses of drugs. **p < 0.01 versus the corresponding control (*t* test). **H** In the colony formation assay, 786-O and Caki-1 cells were treated with increasing concentrations of URB597 and RSL3 singly and in combination for 14 days, and cells were fixed and stained with crystal violet. **I, J** In the wound-healing assay, 786-O and Caki-1 cells were treated with URB597 (10 µM) and RSL3 (0.5 µM) singly and in combination following mitomycin C (1 µM) treatment for the indicated time. Histograms show the relative cell migration (**J**). Scale bar: 100 µm. **p < 0.01 versus the corresponding control (*t* test). Data are means ± SDs of measurements repeated three times with similar results.

Next, we investigated whether FAAH modulates the sensitivity of RCC cells to ferroptosis. FAAH shRNAs were utilized to knockdown of the expression of FAAH in 786-O and Caki-1 cells as determined by western blot **(**Figure S1A). We found that FAAH knockdown strongly sensitized RCC cells to RSL3 (Fig. 2A, B). To explore the synergistic effect of targeting both ferroptosis and FAAH, we next investigated the combinational effect of ferroptosis inducers and various FAAH inhibitors on the growth of 786-O cells with unbiased screening. We found that treatment with both FAAH inhibitors and ferroptosis inducers resulted in greater inhibition compared to treatment with a single agent (Fig. S1B), which motivated us to further investigate the synergic effect on RCC inhibition.

We next assessed the combinational effects across multiple RCC cell lines. As shown in Fig. 2C, URB597 combined with RSL3 significantly triggered cell death in all RCC cell lines tested. In contrast, non-tumorigenic 293T cells were less sensitive to the combinations, which indicates the unique vulnerability of RCC cells to combinational therapies. The dose–response curve for URB597 combined with RSL3 (URB597 + RSL3) revealed high potency and a strong synergistic effect of drug synergy, as determined by the combination index in the Chou–Talalay method (combination index < 1; Fig. 2D, E). Compared to RSL3 alone, URB597 + RSL3 markedly reduced the IC_50_ values of RSL3 in RCC cells (Fig. 2F). In addition, URB597 + RSL3 markedly reduced cell proliferation and colony formation among RCC cells (Fig. 2G, H). These findings demonstrate the highly potent and specific synergy of URB597 and ferroptosis inducers in inhibiting the growth of RCC cells.

Tumor metastasis is the most common cause of mortality due to RCC and remains the greatest challenge in clinical cancer management, so there is an urgent need to develop novel potential candidates to address its progression [48]. Next we examined the synergic effect of RSL3 on migration and invasive capacity in RCC cells. A wound-healing assay showed that RSL3 and URB597 both moderately inhibited the migration and invasion of 786-O and Caki-1 cells. However, combinational treatment had a marked effect on the migration of RCC cells (Fig. 2I, J).

### Synergy of URB597 with RSL3 significantly inhibits the growth of RCC cells by inducing ferroptosis and cell cycle arrest

**Fig. 3:**
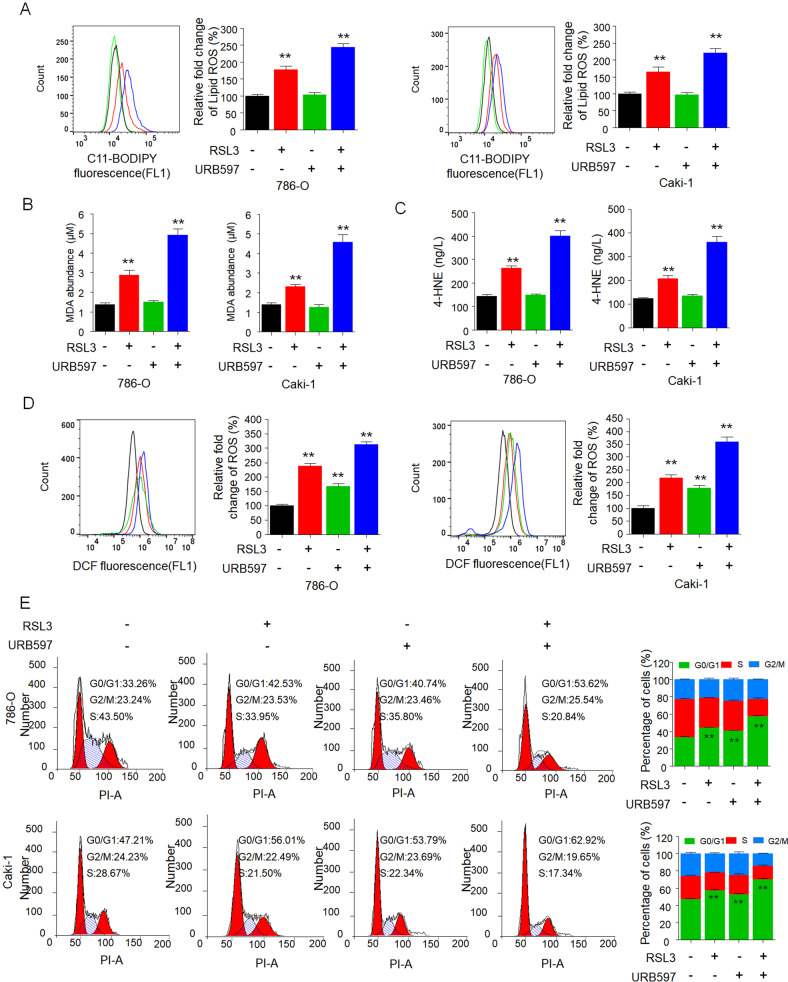
URB597 combined with RSL3 significantly inhibits the growth of RCC cells by inducing ferroptosis and cell cycle arrest. Level of lipid peroxidation (**A**), MDA (**B**), 4-HNE (**C**), and cytosolic ROS production (**D**) in 786-O and Caki-1 cells treated with URB597 (10 µM) and RSL3 (0.5 µM) singly or in combination for 48 h. Lipid peroxidation and cytosolic ROS were assessed by flow cytometry with C11-BODIPY and H_2_DCFDA, respectively. Histograms show the production of MDA, the production of 4-HNE, and the relative fold change in lipid ROS and cytosolic ROS (right panel). **E** Cell cycle distributions of 786-O and Caki-1 cells treated as in (**A**). The cell cycle was evaluated by flow cytometry after staining with propidium iodide (PI). Histograms show the cell cycle distributions of treated 786-O and Caki-1 cells (right). Data are means ± SDs of measurements repeated three times with similar results. **p < 0.01 versus the corresponding control (*t* test).

We next explored the combinational effect on characteristics of ferroptosis, including levels of lipid peroxidation, MDA, 4-HNE, cytosolic ROS promotion in RCC cells. It is intriguing that although URB597 itself did not affect lipid peroxidation, MDA, or 4-HNE levels, it significantly promoted the effect of RSL3 on lipid peroxidation, MDA, and 4-HNE induction compared to treatment with RSL3 alone (Fig. 3A–C). Moreover, combinational treatment markedly increased the cytosolic ROS level compared to the moderate increase for single treatment (Fig. 3D). We also evaluated the synergic effect on the cell cycle in RCC cells. Compared to the modest effect seen with URB597 and RSL3 alone, combinational treatment had a marked effect on inducing G0/G1 cycle arrest (Fig. 3E).

### Inhibition of FAAH regulates sensitivity to ferroptosis by regulating AEA, PEA, and OEA substrates

**Fig. 4:**
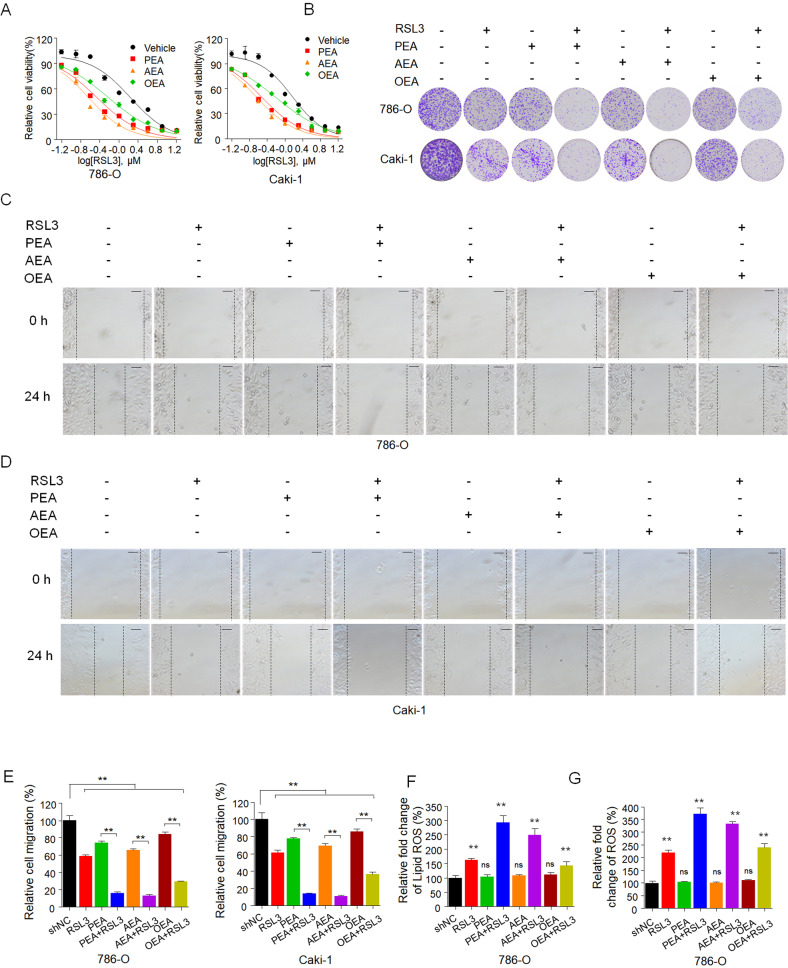
FAAH substrates increase the sensitivity of RCC to ferroptosis inducers. **A** Dose–response curves for RSL3 as a single agent or combined with AEA, PEA, and OEA at the indicated doses in 786-O and Caki-1 cells for 72 h. Viability was measured 72 h after treatment. Effects on cell viability were calculated as the percentage of vehicle-treated cells. **B** In the colony formation assay, 786-O and Caki-1 cells were treated as in (**A**) for 14 days, and cells were fixed and stained with crystal violet. **C–E** In the wound-healing assay, 786-O (**C**) and Caki-1 (**D**) cells were treated as in (**A**) for the indicated time. Histograms show the relative cell migration (**E**). Scale bar: 100 µm. Level of lipid peroxidation (**F**) and cytosolic ROS production (**G**) in 786-O cells treated as in (**A**) for 48 h. Lipid peroxidation and cytosolic ROS were assessed by flow cytometry with C11-BODIPY and H_2_DCFDA, respectively. Histograms show the relative fold change in the production of lipid ROS and cytosolic ROS (right panel). Data are means ± SDs of measurements repeated three times with similar results. ns (not significant), **p < 0.01 (one-way ANOVA).

FAAH is involved in regulating endocannabinoid tone, specifically by decreasing levels of N-acylethanolamides, most important, AEA, PEA, and OEA [49]. We found that AEA, PEA, and OEA significantly enhanced the effect of RSL3 on inhibiting cell colony formation (Fig. 4A, B). We next investigated their combinational effect on the metastasis of RCC cells. We found that AEA, PEA, and OEA significantly enhanced the effect of RSL3 on inhibiting cell migration (Fig. 4C–E). Moreover, they significantly promoted the effect of RSL3 on lipid peroxidation and cytosolic ROS levels compared to the moderate increase seen with RSL3 treatment (Fig. 4F, G).

### RNA-Seq reveals that RSL3 regulates the expression of genes related to cell growth and metastasis

**Fig. 5:**
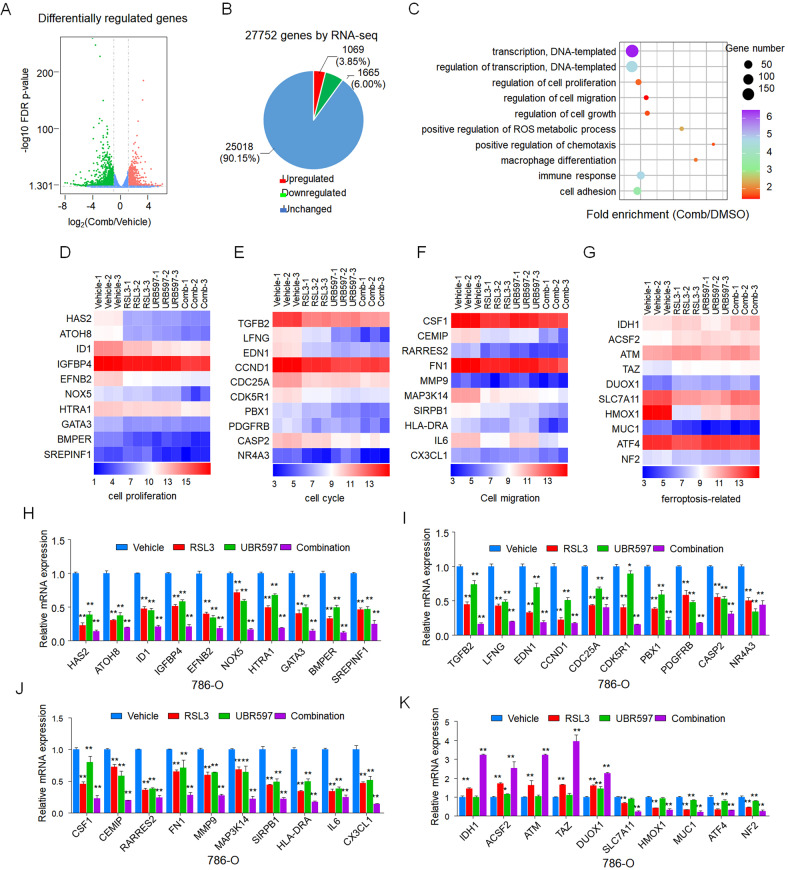
RNA-Seq reveals that the combination of URB597 and RSL3 regulates the expression of genes related to cell growth and metastasis. **A, B** Volcano plot of the results of an RNA-Seq analysis showing the expression of differentially regulated genes in 786-O cells between the control vehicle and combinational treatment (Comb) with URB597 (10 µM) and RSL3 (0.5 µM). Upregulated and downregulated genes are shown in red and green, respectively. Values are presented as the log10 of tag counts. **C** KEGG pathway analysis of differentially expressed genes in transcriptomes of 786-O cells between the control vehicle and combinational treatment (Comb) with URB597 (10 µM) and RSL3 (0.5 µM). The 10 most significantly activated pathways are shown. Heat map of significantly regulated genes in transcriptomes of 786-O cells between the control vehicle and combinational treatment (Comb) with URB597 (10 µM) and RSL3 (0.5 µM) correlated with cell proliferation (**D**), the cell cycle (**E**), cell migration (**F**), and ferroptosis (**G**) (*n* = 3). qPCR analysis of the indicated gene expression associated with cell proliferation (**H**), the cell cycle (**I**), cell migration (**J**), and ferroptosis (**K**) in 786-O cells treated with URB597 (10 µM) and RSL3 (0.5 µM) singly or in combination for 48 h compared to parental 786-O cells. Data are means ± SDs of measurements repeated three times with similar results. ns (not significant), **p < 0.01 versus the corresponding control (*t* test).

To systematically investigate potential mechanisms involved in modulating sensitivity to ferroptosis and the pathway underlying FAAH-regulated ferroptosis, we performed genome-wide RNA-Seq to investigate the impact of combinational treatment on global gene expression (Fig. 5A and Fig. S2A–C). Among the 2734 genes that were significantly differentially expressed, transcripts of 1069 (3.85%) genes were upregulated, whereas transcripts of 1665 (6.00%) genes were downregulated compared to the vehicle (Fig. 5B).

KEGG analysis of the 2734 (9.85%) genes significantly regulated by RSL3 + URB597 demonstrated that combinational treatment modulated signaling pathways, such as the cell proliferation, cell cycle, and cell migration related pathways, which are essential for cancer cell proliferation and metastasis (Fig. 5C–F). It is important to note that various ferroptosis-related genes were also regulated by RSL3 and URB597 (Fig. 5G). Consistent with the RNA-Seq data, qRT-PCR further validated that RSL3 + URB597 had a greater impact on the expression of genes involved in cell proliferation, cell cycle, cell migration, and ferroptosis than either single agent (Fig. 5H–K and Fig. S2D–G).

### The PI3K-AKT pathway is involved in ferroptosis induced by combinational treatment with URB597 + RSL3 in RCC cells

**Fig. 6:**
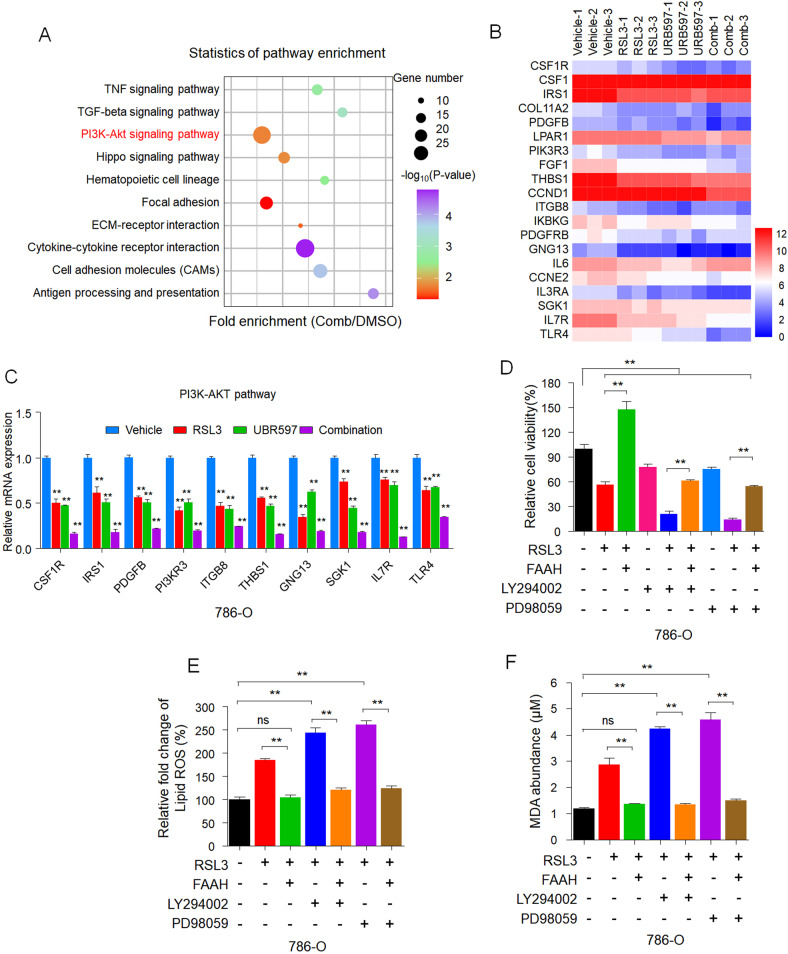
RNA-Seq reveals inhibition of FAAH-regulated sensitivity to ferroptosis via the PI3K-AKT pathway. **A** KEGG pathway analysis of downregulated differentially expressed genes in transcriptomes of 786-O cells after combinational treatment (Comb) with URB597 (10 µM) and RSL3 (0.5 µM). The 10 most significantly activated pathways are shown. **B** Heat map of significantly regulated genes of transcriptomes of 786-O cells after combinational treatment with URB597 (10 µM) and RSL3 (0.5 µM) correlated with the PI3K-AKT pathway (*n* = 3). **C** qPCR analysis of the indicated gene expression associated with the PI3K-AKT pathway in 786-O cells treated with URB597 (10 µM) and RSL3 (0.5 µM) singly or in combination for 48 h compared to parental 786-O cells. **p < 0.01 versus the corresponding control (*t* test). **D** The viability of 786-O cells stably transfected with empty vector or FAAH treated with RSL3 (0.5 µM), LY294002 (5 µM), or PD98059 (10 µM) singly or in combination. Cell viability was assessed by CCK-8 assay after treatment for 72 h with the indicated doses of drugs. Effect of FAAH overexpression, LY294002 (5 µM), and PD98059 (10 µM) on lipid peroxidation (**E**) and MDA (**F**) response to RSL3 treatment in 786-O cells. Data are means ± SDs of measurements repeated three times with similar results. ns (not significant), **p < 0.01 (one-way ANOVA).

To investigate potential mechanisms involved in FAAH-modulated sensitivity to ferroptosis, we compared the KEGG pathway analysis of the 1665 downregulated genes to the vehicle to find that the PI3K-AKT pathway was significantly downregulated (Fig. 6A, B). This indicates that PI3K-AKT pathway contributes to FAAH-regulated sensitivity to ferroptosis.

Consistent with the RNA-Seq data, qRT-PCR further validated that the mRNA level of genes related to the PI3K-AKT pathway was significantly reduced by URB597 and RSL3 singly and in combination (Fig. 6C and Fig. S3A). We next validated whether FAAH modulates sensitivity to ferroptosis by activating AKT and extracellular regulated protein kinases (ERK). Inhibition of ERK1/2 and AKT by PD98059 and LY294002, respectively, enhanced the effect of RSL3 on the viability of 786-O and Caki-1 cells. PD98059 and LY294002 also partially impeded the ability of FAAH to repress the inhibitory effect of RSL3 on decreasing the viability of RCC cells (Fig. 6D and Fig. S3B). Moreover, PD98059 and LY294002 enhanced the effect of RSL3 on lipid peroxidation and MDA production, which were attenuated by FAAH overexpression (Fig. 6E, F and Fig. S3C, D). Taken together, these results show that the PI3K-AKT pathway is involved in ferroptosis induced by combinational treatment with URB597 + RSL3 in RCC cells.

### Synergy of FAAH inhibitors with ferroptosis inducers significantly inhibits RCC tumor growth and metastasis in vivo

**Fig. 7:**
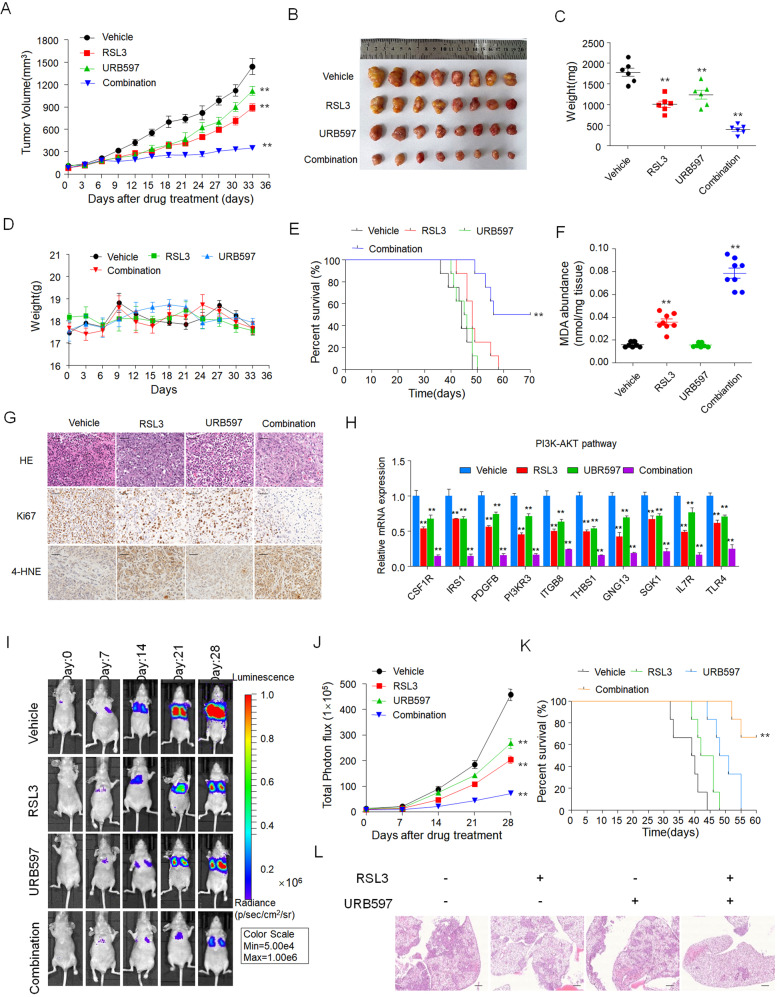
Synergy of URB597 with ferroptosis inducers significantly inhibits RCC tumor growth and metastasis in vivo. 786-O cells were treated with the vehicle, URB597 (30 mg/kg), and RSL3 (30 mg/kg) singly or in combination. Tumor size was monitored every other day (**A**). Photograph of tumors (**B**), tumor weight (**C**), and body weight (**D**) are shown (*n* = 6 per group). **p < 0.01 (*t* test). **E** Survival rates of nude mice treated as in (**A**) (*n* = 6 per group). **p < 0.01 (Kaplan–Meier). **F** Representative images of H&E, Ki67, and 4-HNE IHC staining in tumors harvested from each group. Scale bars: 50 μm. **G** the results of MDA abundance. **H** qPCR analysis of the indicated gene expression associated with the PI3K-AKT pathway in tumors harvested from each group. Data are means ± SDs of measurements repeated three times with similar results. **p < 0.01 versus the corresponding control (*t* test). **I, J** Representative bioluminescent images of animals treated as in (**A**) taken on days 0, 7, 14, 21, and 28 after bioluminescent 786-O xenografts (**I**). Quantification of tumor cells in mice was performed with bioluminescence analysis (**L**) (*n* = 6 per group). **p < 0.01 (*t* test). **K** Survival rates of nude mice treated as in (**A**) (*n* = 6 per group). **p < 0.01 (Kaplan–Meier). **L** Representative images of hematoxylin and eosin staining of paraffin sections from lungs. Scale bar: 100 μm. Data are means ± SDs of six tumors at each time point.

We further examined the combinational effect on 786-O growth in nude mice by assessing the effects of URB597 and RSL3 singly and in combination. URB597 (30 mg/kg) and RSL3 (30 mg/kg) applied as single agents led to modest tumor growth inhibition (TGI)% values of 24.8% and 41.7%, respectively, in 786-O xenograft tumors, whereas the URB597 + RSL3 combination was very effective and drastically reduced tumor sizes, with a TGI% of 82.4% (Fig. 7A–C). This indicates the significant inhibitory effect of the combination treatment on tumor growth.

This drug combination had no obvious toxicity at the applied doses, as indicated by the slight floating body weight during the experiment (Fig. 7D). Kaplan–Meier survival curves of nude mice with 786-O xenograft tumors showed that vehicle treatment, URB597 monotherapy, and RSL3 monotherapy showed no differences in median or durable survival. In contrast, combinational treatment resulted in a significant advantage in durable survival over vehicle and single-agent treatment (Fig. 7E). The median survival of tumor-bearing animals increased with URB597 + RSL3 treatment (63 vs. 44 days), which indicates that URB597 enhances the effect of RSL3 on improving survival. Furthermore, images of hematoxylin and eosin–stained tumor tissue, Ki67-stained tumor tissues, and lipid peroxide 4-HNE-stained immunohistochemical sections of tumors showed that the number of tumor foci was much lower in the combinational treatment group than in the empty-vector or single-agent group, and combination groups showed a more significant inhibitory effect on the amount of Ki67 (proliferation marker) positive cells and significantly increased expression of 4-HNE (Fig. 7F, G). Compared to the vehicle or RSL3 alone, URB597 + RSL3 significantly increased MDA production in tumor tissue, which confirms their profound inhibitory effect on RCC tumor growth via the induction of ferroptosis (Fig. 7F). We also measured AEA, OEA and PEA amounts in the excised tumors by HPLC-MS, which showed that PEA, AEA and OEA levels were increased in tumor samples of mice treated with URB597 and co-treatment with RSL3 and URB597 but not RSL3 single treatment, which confirm the inhibition of FAAH (Figure S3E). Moreover, we found that the mRNA level of genes related to the PI3K-AKT pathway was significantly reduced by URB597 and RSL3 single treatment compared with vehicle group, and combinational treatment exhibited stronger inhibitory effect than single agent (Fig. 7H), which was consistent with in vitro experiments.

To assess the impact of combinational treatment on experimental metastasis, athymic nude mice were given intravenous injections of 786-O cancer cells followed by a 4-week administration of RSL3 and URB597 singly and in combination, respectively. Lung metastasis in the URB597 + RSL3 treatment group was significantly decreased compared to in the single-agent groups (Fig. 7I, J), and survival rates of the URB597 + RSL3 groups were higher than those of the single-agent groups (*P* < 0.05; Fig. 7K). The number of metastatic nodules was significantly reduced in the lungs of animals treated with URB597 + RSL3 (Fig. 7L). Taken together, these results show that synergy of FAAH inhibitors with ferroptosis inducers significantly inhibits RCC tumor growth and metastasis, which demonstrates that targeting both FAAH and ferroptosis could be a promising therapeutic approach to treating RCC.

## Discussion

In this study, we used an unbiased drug combination screen to explore potential synergistic therapies of targeting both FAAH and ferroptosis in RCC cells. We found that the FAAH inhibitor URB597 combined with RSL3 showed potent inhibitory efficiency and high specificity in decreasing the viability of RCC cells via inducing ferroptotic cell death and G1 cell cycle arrest. Knockdown of FAAH enhanced the sensitivity of RCC cells to ferroptosis. Mechanically speaking, through RNA-Seq, we found that URB597 combined with RSL3 had a significant effect on regulating the expression of genes involved in cell proliferation, cell cycle, cell migration, and ferroptosis in RCC cells. KEGG analysis of differentially expressed genes revealed that the PI3K-AKT pathway was involved in ferroptosis induced by combinational treatment with URB597 + RSL3 in RCC cells. Moreover, in vivo evaluation using RCC tumor models revealed the strong synergy of URB597 with RSL3 in decreasing tumor growth and metastasis in RCC. Our results demonstrate that synergy of FAAH inhibitors with ferroptosis inducers could be a promising strategy treating for RCC.

Over the past decades, anticancer effects have been observed for various FAAH inhibitors in several cancer cell lines [35, 36, 50]. One example is colorectal cancer: The FAAH inhibitor AA-5HT (in vitro IC_50_ = 5.6 µM) inhibits the growth and proliferation of Caco-2 cells [51], whereas human colon adenocarcinoma Colo-205 cells show reduced viability, migration, and invasion capabilities after incubation with the FAAH inhibitor PF-3845 (IC_50_ = 2.6 nM) [36, 52]. Similarly, the FAAH inhibitor CAY10401 can reduce cell invasion in LNCaP cells, a prostate carcinoma cell line [33]. Studies have also shown that FAAH inhibitors URB597 and AA-5HT as well as FAAH substrates such as AEA, PEA, OEA, and 2-arachidonic glycerol can inhibit the invasive and metastatic properties of lung cancer cell line A549 in nude athymic mice, in one case even reducing the development of precancerous lesions in mouse colon [53–58].

Importantly, in models of inflammatory pain chronic treatment with FAAH inhibitors does not result in catalepsy and other adverse effects and does not induce tolerance, which is further proof of the safety of these compounds as therapeutic agents [59]. Moreover, clinical trials have shown that in general FAAH inhibitors are well tolerated and exhibit few toxic effects [29]. Indeed, considerable efforts were made in the initial clinical studies to identify selective micromolar/nanomolar FAAH inhibitors such as URB597 with acceptable pharmacokinetic and safety profiles, which do not show similar toxic effects [60]. Here, our results also found that monotherapy with URB597 inhibited the proliferation and metastasis of RCC cells. Although URB597 alone had no effect on ferroptosis, it synergized with RSL3 to significantly reduce cell viability and increase lipid peroxidation compared to monotherapy. Nevertheless, further effort should be made to systematically test the synergy of URB597 with ferroptosis inducers to identify potent inhibitory effects for RCC therapy and the combinational effects of other FAAH inhibitors and ferroptosis inducers.

RNA-Seq of RCC cells treated with URB597 + RSL3 revealed diverse signaling pathways that may regulate ferroptosis sensitivity via PI3K-AKT pathways. Studies have shown that the inhibitory effect of cannabinoid-related drugs on tumor cell proliferation is usually linked to a decrease in the oncogenic PI3K-Akt pathway. A recent study revealed that oncogenic activation of PI3K-AKT-mTOR signaling suppresses ferroptosis via SREBP-mediated lipogenesis [61]. The SREBP related pathway is not only a key regulator of lipid metabolism, but also important therapeutic targets for cancer treatment [62–65]. Here we found that inhibition of ERK1/2 and AKT by PD98059 and LY294002, respectively, enhanced the effect of RSL3 on decreasing the viability of RCC cells as well as lipid peroxidation and MDA production. Apart from this, other signaling pathways, such as the TNF, Hippo, and TGFβ signaling pathways, were also shown to be downregulated as reflected by KEGG analysis. TNF signaling is relevant for cancer tumor progression and metastasis as well as acquired drug resistance [66–68]. TNF antagonists sensitize synovial fibroblasts to ferroptotic cell death in mouse models of collagen-induced arthritis [69]. Dysregulation of the Hippo pathway leads to aberrant activation of YAP and TAZ, the two major transcription coactivators of TEADs, which contributes to apoptosis evasion, cancer development, metastasis, and treatment resistance [70]. YAP/TAZ activation under low density, while conferring resistance to apoptosis, renders cancer cells sensitive to ferroptosis [71–73]. Moreover, dependency on GPX4 exists across diverse therapy-resistant states characterized by high expression of ZEB1, such as TGFβ-mediated therapy resistance in melanoma [74]. In a recent study, TGF-β1 regulated xCT expression, sensitized cells to lipid peroxidation, and ultimately made HCC cell lines vulnerable to a GPX4 inhibitor [75]. Thus, further investigation is needed of the role of TNF, Hippo, and TGFβ signaling in the targeting of both FAAH inhibition and ferroptosis induction.

## Supplementary information

aj-checklist

Supplemental Figure Legend

Supplemental Figure 1

Supplemental Figure 2

Supplemental Figure 3

Original Data of File Supplemental Figure 1A: 786-O-actin

Original Data File of Supplemental Figure 1A: 786-O-FAAH

Original Data File of Supplemental Figure 1A: caki-1-actin

Original Data File of Supplemental Figure 1A: caki-1-actin

## Data Availability

All data generated or analyzed during this study are available from the corresponding author on reasonable request.
