# Supplementary material for: Combination treatment with FAAH inhibitors/URB597 and ferroptosis inducers significantly decreases the growth and metastasis of renal cell carcinoma cells via the PI3K-AKT signaling pathway
Source: Cell Death Dis. 2023 Apr 6;14(4):247. doi: 10.1038/s41419-023-05779-z (PMC10079857; doi:10.1038/s41419-023-05779-z)
Supplement: Supplementary file 2 — Supplemental Figure Legend [file 41419_2023_5779_MOESM2_ESM.docx]

**Supplemental Figure 1 (S1). Synergy screen of FAAH inhibitors and ferroptosis inducers in RCC cells.**

**(A)** Western blotting was conducted to detect FAAH expression in 786-O cells stably transfected with negative control vector (shNC) or shFAAH. **(B)** Combinational effects of FAAH inhibitors (10 µM) and ferroptosis inducers at the indicated concentration in 786-O cells treated with the inhibitors singly or the indicated target pairs. Viability was measured 72 h after treatment with the indicated concentrations of drugs. Effects on cell viability were calculated as the percentage of vehicle-treated cells.

**Supplemental Figure 2 (S2). RNA-Seq reveals that the combination of URB597 and RSL3 regulates the expression of genes related to cell growth and metastasis.**

**(A, B)** A volcano plot of the results of an RNA-Seq analysis showing the expression of differentially regulated genes in 786-O cells between the control vehicle and treatment with URB597 (10 µM) (**A**) and RSL3 (0.5 µM) (**B**). Upregulated and downregulated genes are shown in red and green, respectively. Values are presented as the log10 of tag counts. **(C)** Heat map of a transcriptional profile denoting unbiased clustering of 786-O cells treated with URB597 (10 µM) and RSL3 (0.5 µM) singly or in combination (n = 3). Total RNA was analyzed by high-throughput whole transcriptome sequencing (RNA-Seq). Significant differential expression is defined as an absolute log2 (fold change) ≥ 1 and q < 0.05. **(D–G)** qPCR analysis of the indicated gene expression associated with cell proliferation (**D**), the cell cycle (**E**), cell migration (**F**), and ferroptosis (**G**) in Caki-1 cells treated with URB597 (10 µM) and RSL3 (0.5 µM) singly or in combination for 48 h compared to parental Caki-1 cells. Data are means ± SDs of measurements repeated three times with similar results. ns (not significant), **p < 0.01 versus the corresponding control (*t* test).

**Supplemental Figure 3 (S3). RNA-Seq reveals inhibition of FAAH-regulated sensitivity to ferroptosis via the PI3K-AKT pathway.**

**(A)** qPCR analysis of the indicated gene expression associated with the PI3K-AKT pathway in Caki-1 cells treated with URB597 (10 µM) and RSL3 (0.5 µM) singly or in combination for 48 h compared to parental Caki-1 cells. **(B)** The viability of Caki-1 cells stably transfected with empty vector or FAAH treated with RSL3 (0.5 µM), LY294002 (5 µM), or PD98059 (10 µM) singly or in combination. Cell viability was assessed by CCK-8 assay after treatment for 72 h with the indicated doses of drugs. **(C, D)** Effect of FAAH overexpression, LY294002 (5 µM), and PD98059 (10 µM) on lipid peroxidation **(C)** and MDA **(D)** response to RSL3 treatment in Caki-1 cells. Data are means ± SDs of measurements repeated three times with similar results. ns (not significant), **p < 0.01 (one-way ANOVA). **(E)** PEA, AEA and OEA levels were measured by HPLC-MS in tumor of mice treated the vehicle, URB597 (30 mg/kg), and RSL3 (30 mg/kg) singly or in combination. Data are means ± SDs of measurements repeated three times with similar results. ns (not significant), **p < 0.01 (*t* test).
