# Supplementary figures and images for: Combination treatment with FAAH inhibitors/URB597 and ferroptosis inducers significantly decreases the growth and metastasis of renal cell carcinoma cells via the PI3K-AKT signaling pathway
Source: Cell Death Dis. 2023 Apr 6;14(4):247. doi: 10.1038/s41419-023-05779-z (PMC10079857; doi:10.1038/s41419-023-05779-z)

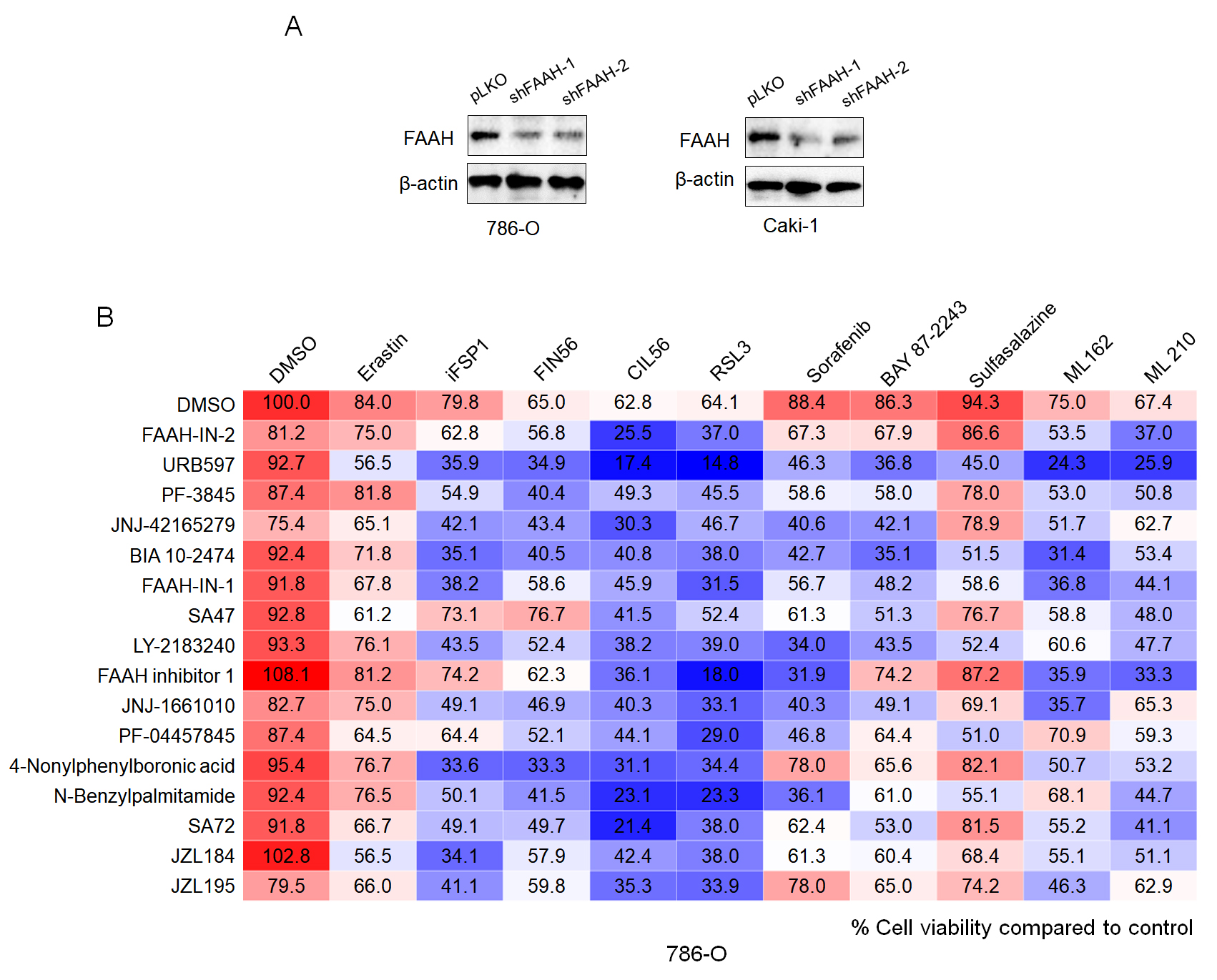

Supplement: Supplementary file 3 — Supplemental Figure 1 [file 41419_2023_5779_MOESM3_ESM.jpg]

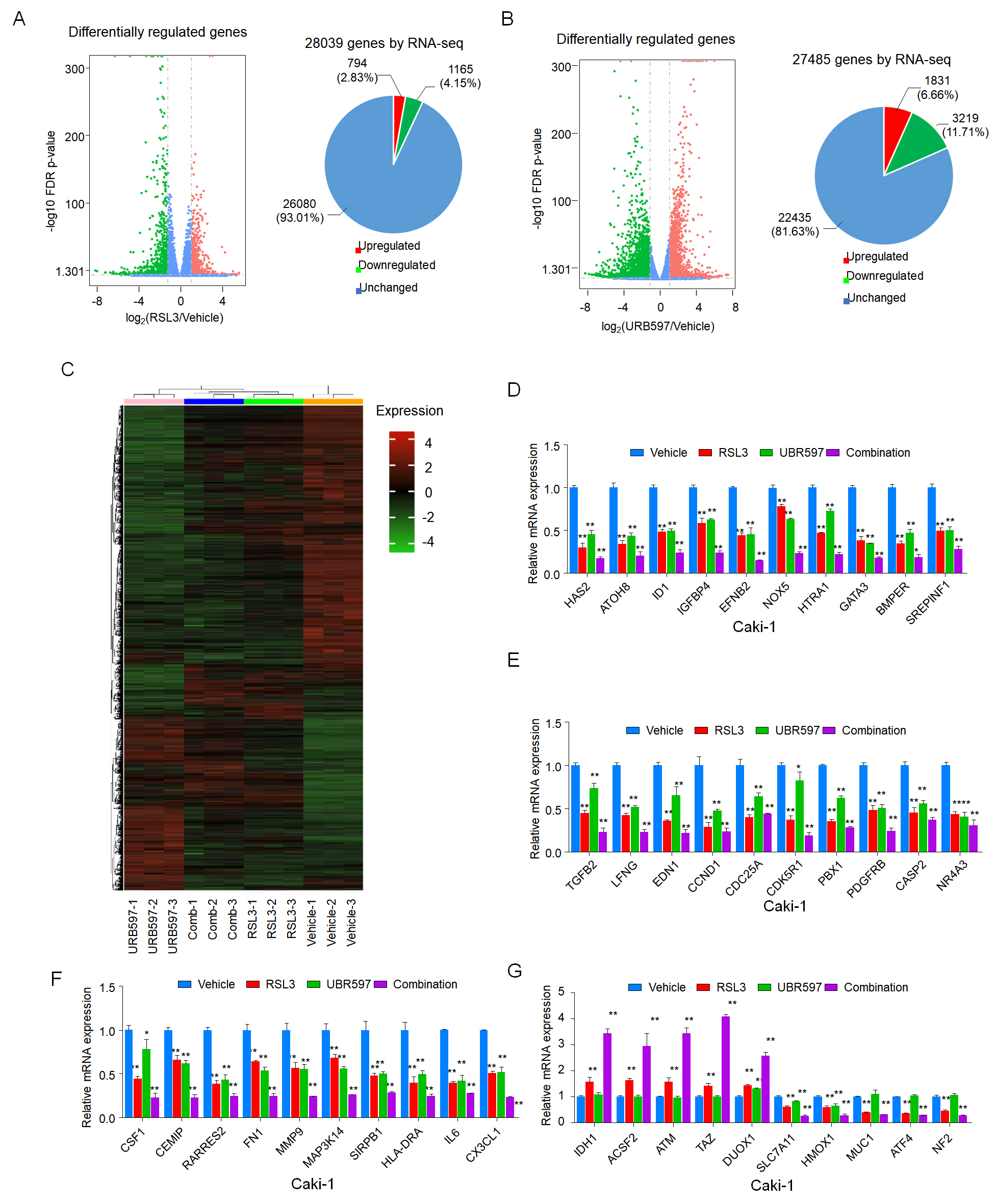

Supplement: Supplementary file 4 — Supplemental Figure 2 [file 41419_2023_5779_MOESM4_ESM.jpg]

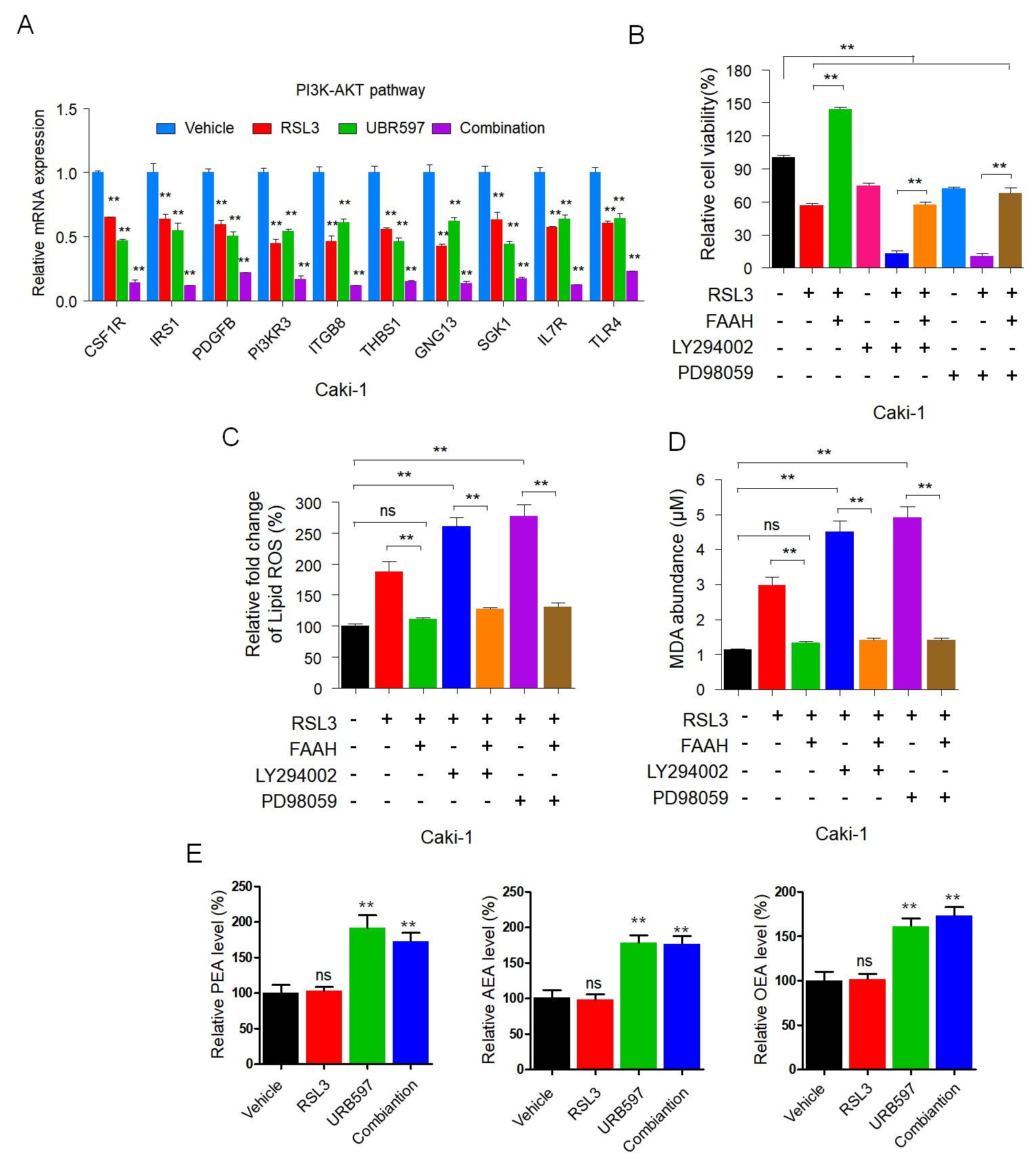

Supplement: Supplementary file 5 — Supplemental Figure 3 [file 41419_2023_5779_MOESM5_ESM.jpg]

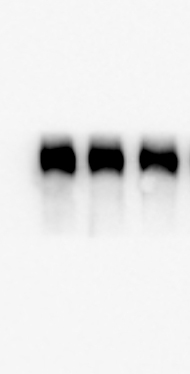

Supplement: Supplementary file 6 — Original Data of File Supplemental Figure 1A: 786-O-actin [file 41419_2023_5779_MOESM6_ESM.jpg]

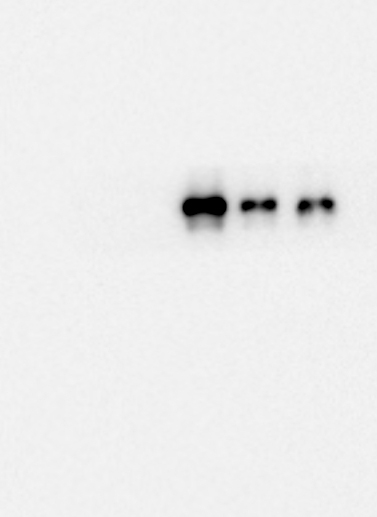

Supplement: Supplementary file 7 — Original Data File of Supplemental Figure 1A: 786-O-FAAH [file 41419_2023_5779_MOESM7_ESM.jpg]

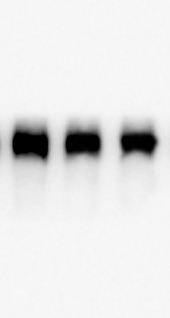

Supplement: Supplementary file 8 — Original Data File of Supplemental Figure 1A: caki-1-actin [file 41419_2023_5779_MOESM8_ESM.jpg]

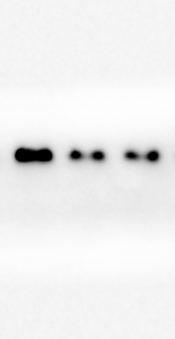

Supplement: Supplementary file 9 — Original Data File of Supplemental Figure 1A: caki-1-actin [file 41419_2023_5779_MOESM9_ESM.jpg]
